# A comparative evaluation of bioequivalence of Gan & Lee glargine U300 and Toujeo^®^ in Chinese healthy male participants

**DOI:** 10.3389/fendo.2024.1407829

**Published:** 2024-08-07

**Authors:** Xiaoli Li, Anshun He, Bingyan Liu, Rongfang Shan, Juan Zhu, Xiaoyue Li, Tian Xie, Yue Li, Mengmeng Chen, He Su, Chaoyang Zhang, Lufeng Li, Dongmei Cheng, Juan Chen, Ying Wang, Yue Su, Yuanyuan Xu, Zhuoran Li, Huan Zhou, Wei Chen, Yuanyuan Liu

**Affiliations:** ^1^ Clinical Trial Center, The First Affiliated Hospital of Bengbu Medical University, Bengbu, China; ^2^ Gan & Lee Pharmaceuticals, Beijing, China; ^3^ School of Pharmacy, Bengbu Medical University, Bengbu, China

**Keywords:** diabetes, insulin glargine U300, bioequivalence, biosimilar, pharmacokinetics, pharmacodynamics

## Abstract

**Background:**

To assess the bioequivalence between Gan & Lee (GL) glargine U300 and Toujeo^®^ regarding pharmacokinetics (PK), pharmacodynamics (PD), and safety in Chinese healthy male participants.

**Methods:**

A single-center, randomized, double-blind, single-dose, two-preparation, two-sequence, four-cycle repeated crossover design study was performed to compare GL glargine U300 and Toujeo^®^ in 40 healthy participants. The primary PK endpoints were the area under the curve of glargine metabolites, M1 concentration from 0 to 24 hours (AUC_0-24h_), and the maximum glargine concentration within 24 hours post-dose (C_max_). The primary PD endpoints were the area under the glucose infusion rate (GIR) curve from 0 to 24 hours (AUC_GIR.0-24h_) and the maximum GIR within 24 hours post-dose (GIR_max_).

**Results:**

GL Glargine U300 demonstrated comparable PK parameters (AUC_0–24h_, C_max_, AUC_0–12h_, and AUC_12–24h_ of M1) and PD responses [AUC_GIR.0–24h_, GIR_max_, AUC_GIR.0–12h_, and AUC_GIR.12–24h_] to those of Toujeo^®^, as indicated by 90% confidence intervals ranging from 80% to 125%. No significant disparities in safety profiles were observed between the two treatment groups, and there were no reported instances of serious adverse events.

**Conclusion:**

The PK, PD, and safety of GL glargine U300 were bioequivalent to that of Toujeo^®^.

**Clinical trial registration:**

https://www.chinadrugtrials.org.cn/, identifier CTR20212419.

## 1 Introduction

Diabetes mellitus, characterized by abnormal insulin secretion, impaired insulin action, or both, is a chronic metabolic disease that results from the interaction of genetic and environmental factors (1). Approximately 537 million people worldwide live with diabetes, with the highest prevalence in China estimated at 140 million (2). The complications of diabetes, which include damage to vital organs such as the heart, kidney, brain, eye, and foot, significantly affect the quality of life of people with diabetes (3, 4). Over the past few decades, numerous insulin analogs have been developed for the treatment of diabetes and to prevent its complications. Long-acting insulin analogs could provide a stable glycemic control for approximately 24 hours with a once-daily injection, greatly simplifying the insulin treatment. This allows for the early initiation of insulin treatment for those who fear needle injections, and early insulin treatment is associated with effective glycemic control with minimal weight gain and hypoglycemia, thus reducing disease-associated complications (5).

Insulin glargine is a human insulin analog produced through recombinant DNA technology. Its unique structure prolongs the decomposition time from hexamer to monomer in clinical application, thus enabling once-daily injection (6). With a well-established efficacy and safety record, insulin glargine is the first long-acting basal insulin analog to be approved for clinical use (Lantus^®^ by Sanofi-Aventis Pharmaceuticals), and it has the highest market share of basal insulin (7). However, hypoglycemia remains a significant challenge for insulin glargine, thereby preventing many patients from achieving optimal glycemic control. In addition, although insulin glargine is commonly prescribed for once-daily injection, some patients may require an additional shot due to diminished efficacy at the end of the dosing interval, which may compromise patient compliance. To overcome the defects of insulin glargine, Toujeo^®^ (insulin glargine U300 or 300 U/mL), an improved and concentrated version of Lantus^®^, was developed with a longer duration of action, stable glucose-lowering profiles, and less day-to-day variability (8).

Toujeo^®^ (Sanofi-Aventis Pharmaceuticals) was first approved in February 2015 by the Food and Drug Administration (FDA) and the European Medicines Agency (EMA) for the treatment of adult diabetes. It is derived from insulin glargine U100 and provides extended 24-hour therapeutic coverage with once-daily administration, thereby ensuring consistent glucose-lowering effects. Compared to the 100 U/mL formulation, the concentrated formulation forms a smaller subcutaneous depot, which allows for a slower and longer-lasting release of insulin (7). This can reduce the required subcutaneous insulin glargine reservoir volume to one-third for insulin glargine U300 compared to U100 (9). As a result, insulin glargine U300 provides more stable and sustained insulin release, slower subcutaneous absorption kinetics, a longer duration of action, and a more stable plasma concentration-time profile (10). Furthermore, insulin glargine U300 exhibits lower intra-day variability and higher intra-day reproducibility than that of the U100 formulation (11). During the dose adjustment phase, individuals treated with Glargine U300 experience a more stable decrease in blood glucose levels (12). Multiple clinical studies have demonstrated that the U300 formulation achieves similar glycemic control to the U100 formulation but with a reduced incidence of clinically significant hypoglycemia and less associated weight gain (13, 14). In addition, individuals treated with insulin glargine U300 have a lower incidence of confirmed daytime and nocturnal hypoglycemia than those treated with insulin glargine U100 (14, 15).

Sanofi was the first pharmaceutical company who launched the insulin glargine U300 in China in November 2020 (branded as Toujeo^®^). As of yet, this remains the only insulin glargine U300 formulation available on the Chinese market. Given the intrinsic advantages of insulin glargine U300 as previously outlined, Gan & Lee (GL) Pharmaceuticals has developed its own insulin glargine U300 injection as a proposed biosimilar to Toujeo^®^. GL Pharmaceuticals is a leading pharmaceutical company in the diabetes industry with about 20 years of experience in manufacturing and marketing insulin glargine U100 (branded as Basalin^®^). GL’s extensive experience in developing Basalin^®^ has facilitated the development of the insulin glargine U300 formulation. It is anticipated that GL insulin glargine U300 will increase the affordability and accessibility of insulin glargine U300, thereby lowering the financial burden for individuals with diabetes. This article presents the results of a phase-I trial conducted in healthy Chinese male participants to evaluate the bioequivalence of GL insulin glargine U300 (test product, T, Gan & Lee Pharmaceuticals, 3mL: 900U) to Toujeo^®^ (reference product, R, Sanofi, 1.5mL: 450U).

## 2 Methods

### 2.1 Study design

In this phase I, single-center, double-blind, randomized trial with a single-dose, two-preparation, two-sequence, four-cycle repeated crossover design, forty eligible participants were evenly randomized to the TRTR group and the RTRT group to receive either the test or the reference product by using a method of random number table. The investigator and subjects remained masked to treatment assignment throughout the study. This study was conducted in accordance with the guidelines on good clinical practice and in compliance with the Declaration of Helsinki. The study was approved by the Ethics Committee of the First Affiliated Hospital of Bengbu Medical University.

An overview of the study design is shown in **Supplementary Figure 1**. The screening period spans from 28 days prior to the start of the study until 1 day prior to the start. The treatment will be administered once per cycle, for a total of 4 cycles, with a washout period of 14-21 days between each cycle to avoid any carryover effect in a crossover design. During each treatment cycle, pharmacokinetics (PK) and pharmacodynamics (PD) were assessed using a euglycemic glucose clamp. A follow-up will be conducted on the 8th day (± 1 day) after the last dose.

A euglycemic glucose clamp was conducted using the ClampArt^®^ device, which continuously monitored the subject’s blood glucose and administered glucose infusion rates (GIR) to maintain blood glucose at a concentration as close as possible to the target blood glucose concentration. More details regarding the clamp methodology were provided in the **Supplementary Materials**.

### 2.2 Study participants

All participants provided written informed consent prior to randomization. The clinical trial was conducted in the First Affiliated Hospital of Bengbu Medical University. Chinese healthy males aged 18-45 years with a body mass index (BMI) of 19-24 kg/m^2^ and a body weight of at least 50 kg were recruited in this study. Detailed inclusion and exclusion criteria can be found in the **Supplementary Materials**.

### 2.3 Study endpoints

The primary PK endpoints were the area under the insulin glargine concentration curve from 0 to 24 hours (AUC_0-24h_) and the maximum observed insulin glargine concentration within 24 hours post-dose (C_max_). The primary PD endpoints were the area under the glucose infusion rate curve from 0 to 24 hours (AUC_GIR.0-24h_) and the maximum glucose infusion rate within 24 hours post-dose (GIR_max_). Safety endpoints included adverse events (AEs), serious adverse events (SAEs), laboratory safety, physical examinations, vital signs, electrocardiograms (ECGs), and local tolerability at the injection site. Detailed information on the secondary PK/PD endpoints was provided in the **Supplementary Materials**.

### 2.4 Data statistics

Statistical analyses were conducted using SAS 9.4 and WinNonlin 8.2 software. Details of the sample size calculation are provided in the **Supplementary Materials**. To assess the quality of the clamp test, descriptive statistics were performed on the mean, standard deviation, and coefficient of variation of blood glucose and C-peptide following each cycle of test administration for each participant. Descriptive statistics were performed on the PK/PD parameters of insulin glargine. For the main metabolites M1 and M2, the values of C_max_ and Time to peak drug concentration (T_max_), maximum concentration (C_max_), and AUC_0-24h_ were transformed by natural logarithm, and inter-subject and inter-period linear mixed effect model analysis was performed. Subsequently, the 90% confidence interval (CI) was calculated using a double one-sided t-test, and a non-parametric test statistical analysis method was used to evaluate and judge the PK characteristics of the drug and its bioequivalence.

Key PD metrics, GIR_max_ and T_GIRmax_, were evaluated. Natural logarithms of GIR_max_ and AUC_GIR.0-24h_ were taken, followed by inter-subject and inter-period linear mixed-effect model analyses. The 95% CI was determined using a two-sided t-test, with non-parametric methods employed to assess the drug’s PD properties and bioequivalence.

Bioequivalence valuation was performed with PK parameters (C_max_, AUC_0-24h_) and PD parameters (GIR_max_, AUC_GIR.0-24h_) of the analyses as indicators. The intra-subject coefficient of variation (CV_wr_) of the PK parameters and PD parameters of the reference product was initially calculated. If CV_wr_ < 30%, bioequivalence assessment used the average bioequivalence (ABE) method, with a target range of 80.00%~125.00%. For CV_wr_ ≥ 30%, the reference product was subjected to the reference-scaled average bioequivalence (RSABE) method.

Adverse events occurred in this study were described according to MedDRA System Organ Classification (SOC) and preferred term (PT). The incidence and severity of adverse events, and the relationship between adverse events and the investigational drugs were described statistically.

## 3 Results

### 3.1 Description of the study population

A total of 232 participants were screened, of which 40 participants were enrolled, and 36 participants completed the study (**Supplementary Figure 2**). Both the full analysis set (FAS) and safety data analysis set (SS) included 40 participants. The pharmacokinetics analysis parameter set (PKPS) and pharmacodynamics analysis parameter set (PDPS) included 39 participants. The baseline demographic characteristics of the participants in the TRTR and RTRT groups were comparable, with a mean age of 28 ± 5 years and 27 ± 5 years, a mean weight of 64.6 ± 5.3 kg and 63.6 ± 5.9 kg, a mean BMI of 22.1 ± 1.0 kg/m^2^ and 21.8 ± 1.6 kg/m^2^, and a mean blood potassium level of 3.88 ± 0.35 mmol/L and 3.81 ± 0.31 mmol/L, respectively (**Supplementary Table 1**).

### 3.2 Pharmacokinetics

**Figure 1:**
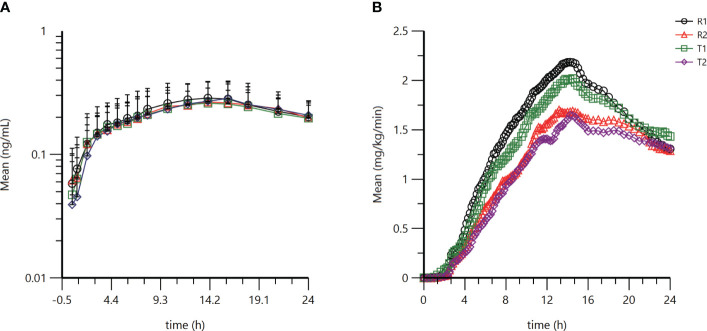
Pharmacokinetic and pharmacodynamic profiles of the test product and reference product. **(A)** Semilogarithmic mean blood concentration-time curve of M1 after subcutaneous injection of test product T or reference product R **(B)** Average GIR-time curve after subcutaneous injection of test product T or reference product R. Analysis was performed using SAS 9.4.

PK profiles for the metabolite M1 of glargine were similar between the test and the reference product (**Table 1**). The geometric mean ratios (GMR) of C_max_ and AUC_0-24h_ for the M1 of the test product T and the reference product R were 100.32% and 98.64%, respectively. The 90% CI of the GMR of C_max_ and AUC_0-24h_ of the active metabolite M1 of the test product T and the reference product R were 92.78% to 108.47% and 90.94% to 106.99%, respectively, which fell within the biological equivalence range for efficacy (80.00% to 125.00%) (**Table 2**). The results indicated that the test product was equivalent to the reference product in terms of PK endpoints (**Figure 1A**).

**Table 1:** PK parameters (M1) and PD parameters of the test product and reference product.

	Test product	Reference product
PK parameters		
C_max_ (ng/ml)	0.303 ± 0.102	0.307 ± 0.118
T_max_ (h)	14.64 ± 4.10	14.91 ± 3.89
AUC_0-24h_ (h*ng/ml)	4.99 ± 1.65	5.16 ± 2.01
AUC_0-12h_ (h*ng/ml)	2.07 ± 0.803	2.19 ± 1.02
AUC_12-24h_ (h*ng/ml)	2.91 ± 0.919	2.96 ± 1.05
t_1/2_ (h)	16.6 (12.78, 27.73)	15.6 (11.85, 31.35)
PD parameters		
T_GIRmax_ (h)	14.65 ± 3.65	13.79 ± 3.81
GIR_max_ (mg kg^-1^ min^-1^)	2.04 ± 0.97	2.14 ± 1.06
AUC_GIR.0-24h_ (h*mg kg^-1^ min^-1^)	28.1 ± 15.0	29.9 ± 15.5
AUC_GIR.0-12h_ (h*mg kg^-1^ min^-1^)	8.84 ± 7.29	9.88 ± 6.90
AUC_GIR.12-24h_ (h*mg kg^-1^ min^-1^)	19.2 ± 8.44	20.0 ± 9.30

Data were presented as Mean ± SD or Median (Lower quartile, Upper quartile).

**Table 2:** C_max_ and AUC_0-24h_ of M1 after subcutaneous injection of test product T and reference product R by participants after logarithmic transformation (1-2α) confidence interval test -ABE method.

Parameters	Geometric mean ratio			CV_wr_ (%)	90% CI (%)
	Test product (T)	Reference product (R)	T/R (%)		
**C_max_ (ng/mL)**	0.29	0.29	100.32	26.2	92.78~108.47
AUC_0-24h_ (h* ng/mL)	4.72	4.79	98.64	24.4	90.94~106.99

### 3.3 Pharmacodynamics

After receiving either the test product or the reference product, the mean C-peptide plasma concentration to basal C-peptide plasma concentration ratio was determined to be 0.6 (**Supplementary Figure 3**, **Supplementary Table 2**). Additionally, the participants’ blood glucose levels were successfully maintained within the target range, and endogenous insulin secretion was effectively suppressed (**Supplementary Figure 4**, **Supplementary Table 3**). These findings indicate the consistent quality of the clamp test in this study, and the data obtained can be utilized for the analysis of the PK and PD characteristics of the investigational drugs.

GIR profiles between the two treatments were comparable (**Table 1**). The GMR for GIR_max_ and AUC_GIR.0-24h_ between test product T and reference product R were 96.86% and 94.10%, respectively (**Table 3**). The CV_wr_ of GIR_max_ and AUC_GIR_ of the test product T and the reference product R were 31.0% and 39.7% (both ≥ 30%), and the equivalence assessment was performed using the RSABE method. The 95% upper confidence interval bound values of the GIR_max_ and AUC_GIR_ were -0.0483 and -0.0749 (both ≤ 0), and the point estimates of the GMR of the T and R products were 0.9609 and 0.9329, respectively, meeting the bioequivalence criteria of 0.80 and 1.25. The GIR_max_ and AUC_GIR.0-24h_ of the test product T and the reference product R are bioequivalent (**Table 3**). Overall, the results demonstrate that the test product is equivalent to the reference product in terms of the PD endpoints (**Figure 1B**).

**Table 3:** GIR_max_ and AUC_GIR_ of participants after subcutaneous injection of test product T and reference product R, RSABE method.

Parameters	Geometric mean ratio			CV_wr_(%)	Point estimate
	Test product (T)	Reference product (R)	T/R (%)		
**GIR_max_ (mg kg^-1^ min^-1^)**	1.87	1.93	96.86	31.0	0.9609
AUC_GIR.0-24h_ (h*mg kg^-1^ min^-1^)	24.84	26.40	94.10	39.7	0.9329

### 3.4 Safety

Both test product (GL glargine U300) and reference product were well tolerated, with comparable proportions of participants reporting AEs (T1: 51.3%, T2:41.7%, R1: 40.0%, R2: 47.4%, **Table 4**) and drug-related AEs (T1: 33.3%, T2:30.6%, R1: 20%, R2: 18.4%, **Table 4**). The incidence of AEs was notably consistent across both test and reference products, indicating a comparable safety profile in the context of initial and repeated exposures.

**Table 4:** Overview of adverse events.

	Test product (T1) (N=39)		Test product (T2) (N=36)		Reference product (R1) (N=40)		Reference product (R2) (N=38)	
	Numbers	Subjects (%)	Numbers	Subjects (%)	Numbers	Subjects (%)	Numbers	Subjects (%)
AEs	31	20 (51.3)	28	15 (41.7)	27	16 (40.0)	35	18 (47.4)
Drug-related AEs	15	13 (33.3)	15	11 (30.6)	9	8 (20.0)	11	7 (18.4)
SAEs	1	1 (2.6)	0	0 (0.0)	2	1 (2.5)	0	0 (0.0)
Drug-related SAEs	0	0 (0.0)	0	0 (0.0)	1	1 (2.5)	0	0 (0.0)
AEs leading to discontinuation	2	2 (5.1)	0	0 (0.0)	1	1 (2.5)	3	2 (5.3)
Drug-related AEs leading to discontinuation	0	0 (0.0)	0	0 (0.0)	0	0 (0.0)	0	0 (0.0)

N, number of participants in each group. Note: The percentage is calculated using the number of participants in each group as the denominator. Adverse events refer to those that are definitely related, likely to be related, or possibly related to the drug. AEs, Adverse events, SAEs, Serious adverse events.

During each dosing period, the predominant AEs were laboratory abnormalities and hematologic/lymphoid disorders. Most AEs were mild and all resolved, with the exception of one participant who was lost to follow-up. SAEs included tuberculous pleurisy, hypokalemia, and seizures. Notably, both the test and reference products exhibited similar AE incidence and severity, suggesting comparable safety profiles. Four participants discontinued the study due to AEs that were not drug-related. Two (5.1%) developed tuberculous pleurisy and device-associated infection during the first period of test product injection. One (2.5%) experienced seizures during the first period, and two others (5.3%) developed device-associated infections, deep vein thrombosis, and venous thrombosis of the extremities during the second period of reference product injection (**Supplementary Tables 4–6**).

## 4 Discussion

This clinical trial demonstrated comparative PK and PD profiles between GL insulin glargine U300 and Toujeo^®^ through the utilization of the glucose clamp technique.

The euglycemic clamp technique is a standard approach for assessing insulin activity and demonstrating biosimilarity between insulin products in clinical pharmacology studies (16, 17). In this trial, the clamp duration was 24 hours, allowing for a comprehensive assessment of the complete PK and PD profiles of long-acting basal insulin following a single dose. Per the guideline recommendation of the FDA and EMA, the dose of 0.4 U/kg was selected as it is anticipated to produce a robust dose-response relationship and reduce the variability among study participants (18, 19). A randomized and double-blind method was utilized to minimize potential biases arising from associations between the allocation order of the investigational products and subject characteristics. Additionally, to prevent any carryover effects between dose administrations, a minimum resting period of 14-21 days was enforced between consecutive dosing visits.

The present study employed a crossover design, which is recommended for bioequivalence studies to mitigate bias from inter-subject variation effectively (20). Moreover, cross-group designs are more efficient than parallel-group designs when the sample sizes are equal. Crossover designs with replication typically require smaller sample sizes than 2 × 2 crossover designs and allow estimation of intra-subject variability for both test and reference formulations. Prior studies of insulin glargine have shown that intra-subject variability in PK parameters is at least 35%, which has resulted in decreased precision (21–23). Therefore, a two-preparation, two-sequence, four-cycle repeated crossover design control study was carried out in this trial with the objective of reducing variability and enhancing statistical power. This approach allowed each participant to serve as their own control, thereby reducing the required number of participants compared to a parallel group design. Nevertheless, a total of 40 healthy male Chinese participants were enrolled to ensure adequate test performance and to account for potential dropouts.

Previous clinical PK/PD comparative studies of insulin glargine have reported an intra-subject variability of PK parameters (AUC_0-24h_ and C_max_) at approximately 30.0%, with even greater intra-subject variability observed for PD parameters (AUC_GIR.0-24h_ and GIR_max_) (24). For the reference product Toujeo^®^, intra-subject variability for AUC_0-24h_ and C_max_ was 21% and 26%, respectively, while intra-subject variability for AUC_GIR.0-24h_ and GIR_max_ was 40.3% and 41.3%, respectively (25). This indicates that insulin glargine U300 exhibits considerable variability in its response. These findings were further corroborated by the results of the present clinical trial. During the PK analysis of the present clinical trial, the intra-subject variability for C_max_ and AUC_0-24h_ of insulin metabolite M1 remained below 30%, thus justifying the use of the ABE method for the bioequivalence assessment. In contrast, the RSABE method is recommended when the intra-subject coefficient of variation exceeds 30% for one or more PK or PD parameters (26). The PD bioequivalence is assessed using the RSABE method, given that the CV_wr_ for PD parameters GIR_max_ and AUC_GIR.0-24h_ are 31.0% and 39.7%, respectively, both greater than 30%.

Upon the subcutaneous injection in humans, insulin glargine is rapidly metabolized into M1 and M2. These metabolites exhibit the same metabolic properties as insulin glargine and serve as indicators of the active drug’s concentration in plasma (27). Previous studies have established that insulin glargine and M2 are generally undetectable, making M1 concentrations the key parameter for calculating PK endpoints and assessing bioequivalence (21, 28, 29). During the present study, the M1 PK profiles remained highly consistent over the 24-hour assessment period. While M2 was also collected and evaluated in this study, a substantial amount of M2 data from participants was missing due to concentrations below the lower limit of detection, making it impossible to use the M2 data to assess the PK/PD similarity between the two preparations.

The euglycemic glucose clamp is a well-established method for assessing the PD profile of insulin products and comparing their pharmacologic similarity. A high-quality clamp study typically exhibits a coefficient of variation in blood glucose concentration of less than 5.0% and a mean difference within 5.0% (24). The present study confirmed the high clamp quality based on metrics that characterize accuracy and precision relative to the clamp target glucose concentration. In this study, the coefficient of variation for blood glucose in the test preparation group was 6.0%, while in the reference preparation group, it was 6.3%, which is slightly above the desired threshold of less than 5%. Nevertheless, both groups displayed similar coefficients of variation in blood glucose. Furthermore, the post-administration blood glucose concentrations were controlled within ± 10% of the target range in both groups, exhibiting very close similarities (4.18 ± 0.25 for T versus 4.18 ± 0.26 for R). In addition, both groups experienced suppression of endogenous insulin secretion (mean C-peptide post-administration/basal C-peptide is 0.6 ± 0.1 for both products) during the clamp period, which further supports the high quality of the clamp.

The safety profiles of the test and reference products were comparable, with no significant differences in the incidence or severity of adverse events. Following injections of the test product (T1 and T2) and reference product (R1 and R2), AE incidences were similar between the two groups. In summary, the safety profile of GL insulin glargine U300 did not raise any concerns and was similar to that of the reference product. However, the present study did not include any immunogenicity assessments of the investigational product, which could be a potential flaw. Although the clinical immunogenicity studies may be waived in certain cases, the local regulatory authority may require additional evaluation of the GL product’s immunogenicity (18, 19).

## 5 Conclusion

The PK, PD, and safety of Gan & Lee insulin glargine U300 were comparable to those of Toujeo^®^. This study provides critical clinical evidence that GL insulin glargine U300 is a proposed biosimilar.

## Data availability statement

The original contributions presented in the study are included in the article/**Supplementary Material**. Further inquiries can be directed to the corresponding author.

## Ethics statement

The studies involving humans were approved by The First Affiliated Hospital of Bengbu Medical University. The studies were conducted in accordance with the local legislation and institutional requirements. The participants provided their written informed consent to participate in this study.

## Author contributions

XLL: Investigation, Data curation, Writing – review & editing. AH: Writing – original draft, Writing – review & editing. BL: Investigation, Data curation, Writing – review & editing. RS: Investigation, Writing – review & editing, Data curation. JZ: Writing – review & editing, Investigation, Data curation. XYL: Writing – review & editing, Investigation, Data curation. TX: Writing – original draft, Writing – review & editing. YL: Writing – review & editing, Writing – original draft. MC: Writing – review & editing, Investigation, Data curation. HS: Writing – review & editing, Investigation, Data curation. CZ: Writing – review & editing, Investigation, Data curation. LL: Writing – review & editing, Investigation, Data curation. DC: Writing – review & editing, Investigation, Data curation. JC: Writing – review & editing, Investigation, Data curation. YW: Writing – review & editing, Investigation, Data curation. YS: Writing – review & editing, Investigation, Data curation. YX: Writing – review & editing, Investigation, Data curation. ZL: Writing – review & editing, Project administration, Data curation. HZ: Writing – review & editing, Supervision, Project administration. WC: Writing – review & editing, Supervision, Project administration. YYL: Writing – review & editing, Supervision, Project administration.
