# Supplementary material for: A comparative evaluation of bioequivalence of Gan & Lee glargine U300 and Toujeo® in Chinese healthy male participants
Source: Front Endocrinol (Lausanne). 2024 Aug 7;15:1407829. doi: 10.3389/fendo.2024.1407829 (PMC11337195; doi:10.3389/fendo.2024.1407829)
Supplement: Supplementary file 1 [file DataSheet_1.docx]

Supplementary Material

# Inclusion criteria

To be selected, subjects must meet all the following criteria:

1) Voluntary participation and signing of the informed consent form (ICF).

2) Healthy Chinese men aged between 18 and 45 years old (inclusive).

3) Body mass index (BMI) at screening should be between 19.0 and 24.0 kg/m^2^ (inclusive),

4) Weight should be equal to or greater than 50.0 kg.

5) No family history of obesity and diabetes.

6)According to the “China Guidelines for the Prevention and Treatment of Type 2 Diabetes (2017 Edition)”, normal glucose tolerance is defined as fasting blood glucose levels between 3.9 mmol/L and 6.1 mmol/L, and endpoint tolerance test (OGTT) blood glucose levels 2 hours after taking sugar below 7.8 mmol/L. The functioning of the islands has been verified by the Island Release Test (IRT).

7) The glycated hemoglobin level should be less than or equal to 6%.

8) Sufficient intravenous access is necessary to establish blood collection channels according to the trial protocol.

9) Participants must not have any birth plans during the study and must agree to use reliable contraceptive measures until 6 months after the last dose of the experimental drug. They must also have no plans for sperm donation.

10) Participants must be able to communicate effectively with researchers and comply with research regulations.

# Exclusion criteria

Subjects who meet one of the following conditions will be excluded:

1) During screening, history of any past or current medical conditions related to the heart, lung, liver, kidney, pancreas, digestive tract, nervous system, and endocrine system. This includes but is not limited to blood disorders, metabolic disorders, tumor, cancer, immune, and mental disorders, as well as cardiovascular and cerebrovascular diseases.

2) The researcher identified abnormal and clinically significant vital sign, physical examination, laboratory test, chest X-ray, and 12-lead electrocardiogram results during screening.

3) History of hypoglycemia within 3 months prior to screening.

4) Individuals with recent serious infection or trauma within 4 weeks prior to screening or surgery within 3 months prior to screening.

5) Participants with severe allergies (such as allergies to more than 3 allergens, allergic asthma affecting the lower respiratory tract, or allergies requiring glucocorticoid treatment) or with a known history of allergies to the experimental drug ingredients.

6) History of drug intake within 4 weeks before screening that affects the hypoglycemic effect of insulin, such as corticosteroids, danazol, diazoxide, diuretics, epinephrine, albuterol, terbutaline, glucagon, or growth hormone. Additionally, thyroid hormone and beta-blockers should also be avoided.

7) Individuals who test positive for hepatitis B surface antigen (HBsAg), Treponema pallidum antibody (EIA), AIDS antibody (Anti-HIV), and hepatitis C virus antibody (Anti-HCV).

8) Use of prescription drugs, Chinese herbal medicines, over-the-counter drugs, or health products (excluding routine vitamin supplements) within 2 weeks prior to screening.

9) Within the past 6 months prior to screening history of blood donation; a total blood loss of at least 200 ml due to any reason; blood transfusion or use of any blood products.

10) Those who are judged by the researcher to have any type of anemia.

11) Those who smoked more than 5 cigarettes per day on average within 3 months before screening, or who do not agree to refrain from smoking during the trial.

12) Drinking more than 14 units per week in the 3 months before screening: 1 unit ≈ 360 ml of beer, or 45 ml of spirits, or 150 ml of wine. Drinking within 48 hours before using the test drug or not abstaining from alcohol during the test or those who have a positive alcohol breath test result during screening.

13) Individuals with a history of drug abuse within 6 months prior to screening or a positive urine test for illegal drugs (morphine, methamphetamine, ketamine, methylenedioxyamphetamine, tetrahydrocannabinolic acid) during screening.

14) Individuals who have made significant changes to their diet or exercise habits within 3 months prior to screening, such as those who engage in strenuous exercise.

15) The participant's eligibility for this clinical trial will be affected if they have used investigational drugs or medical devices within three months prior to screening.

16) Individuals with food allergies or special dietary requirements that prevent adherence to a standardized diet (such as intolerance to standard meals, lactose intolerance, etc.).

17) Individuals with a history of fainting during needle and blood procedures, those who cannot tolerate venipuncture blood collection, and/or those who have difficulty with blood collection.

18) Individuals who develop acute illness during the screening period.

19) Individuals who are deemed unsuitable to participate in this trial by the researcher's evaluation.

# Secondary PK/PD endpoints

Secondary PK endpoints included the AUC from 0 to 12 hours (AUC_ins.0-12h_) and the AUC from 0 hours to infinity (AUC_ins.0-inf_). Secondary PD endpoints included the area under the glucose infusion rate curve from 0 to 12 hours (AUC_GIR.0-12h_) and the time to maximum glucose infusion rate (T_GIR.max_).

# Clamp methodology

A euglycaemic glucose clamp was conducted using the ClampArt^®^ device. Participants fasted overnight starting at 8:00 p.m. before each cycle. After a vascular assessment conducted by investigators, a central venous catheter was used for the infusion of 20% glucose solution in one upper limb, while a needle was positioned in the opposite limb for blood sampling. During the test, the participant’s limb was warmed using an electric cuff at 50-60°C to arterialize venous blood. Blood glucose was measured at intervals (-30, -20, -10, and -5 min) prior to insulin glargine injection, with the average of these readings serving as the baseline. Insulin glargine U300 (0.4 U/kg) was then administered subcutaneously near the umbilicus. During the 24 hours clamp study, the glucose infusion rate (GIR) was adjusted based on blood glucose readings to keep levels within ±10% of the target, set at 0.3 mmol/L below baseline.

# Sample size determination

The sample size is determined by referring to the original drug's instructions of this product. According to the 2*4 replicated crossover study design, the converted pharmacokinetic/ pharmacodynamic parameters C_max_, AUC_0-24h_, GIR_max_, AUC_GIR.0-24h_ were used as the main analytical indices and it was assumed that α=0. 05, power=80%, intra-CV=20%, GMR=90%, bioequivalence interval=80.00%~125.00%, and some degree of shedding was taken into account. A total of 40 healthy Chinese male subjects were expected to be enrolled in the study.

# Supplementary Figures and Tables

## Supplementary Figures


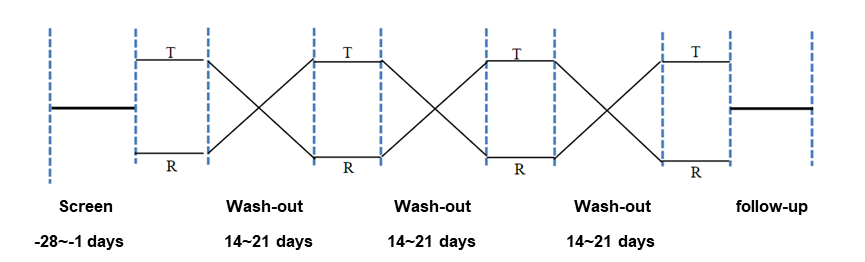


**Supplementary Figure 1.** An overview of the study design. The total duration of this study about 67-115 days. The safety telephone follow-up was conducted at 8 ± 1 days after the last administration.


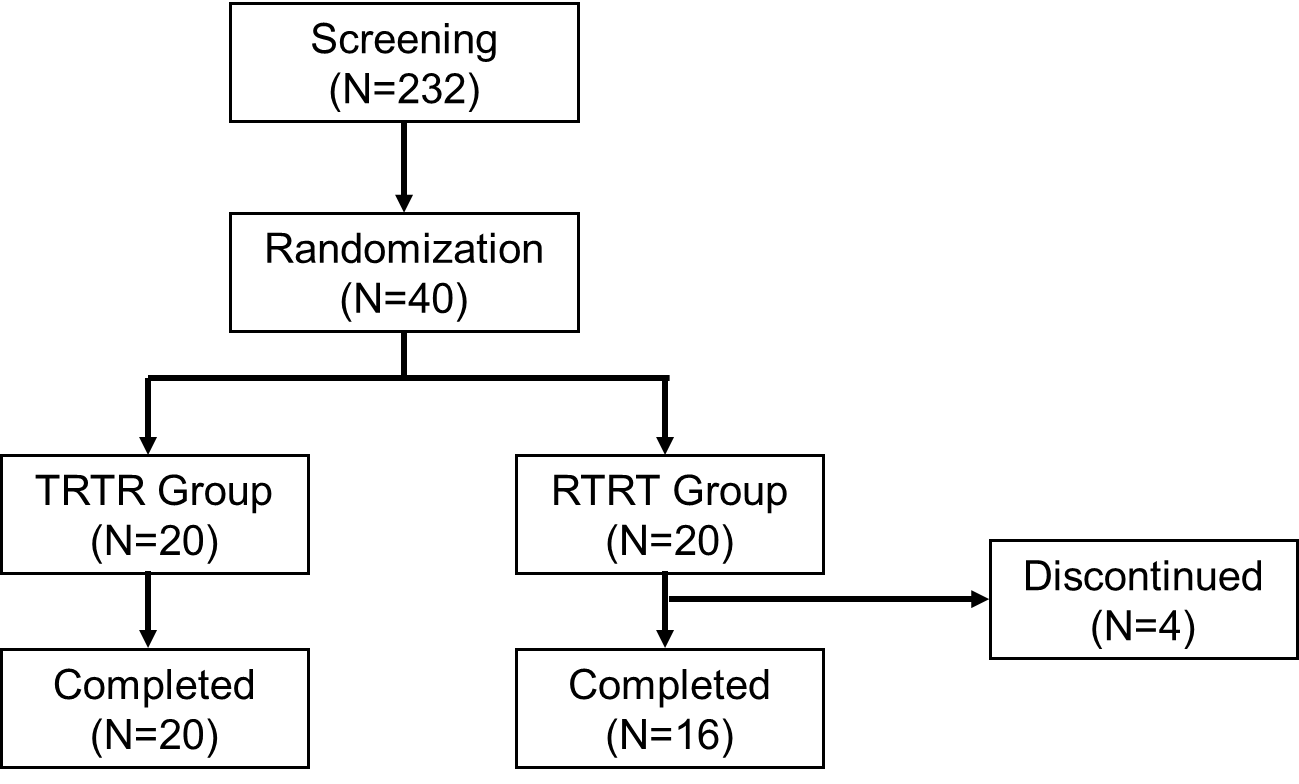


**Supplementary Figure 2.** An overview of the disposition. Four participants discontinued the study. The investigator determines that the subject is experiencing factors that make continuation of the trial inappropriate and actually terminates the subject's continued participation in the trial.


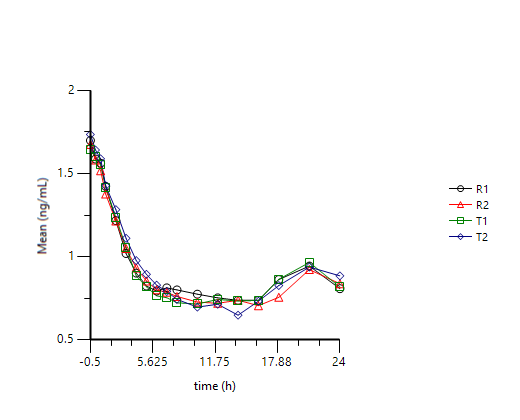


**Supplementary Figure 3.** Average C-peptide concentration-time curves before and after subcutaneous injection of test product T or reference product R in 39 subjects.


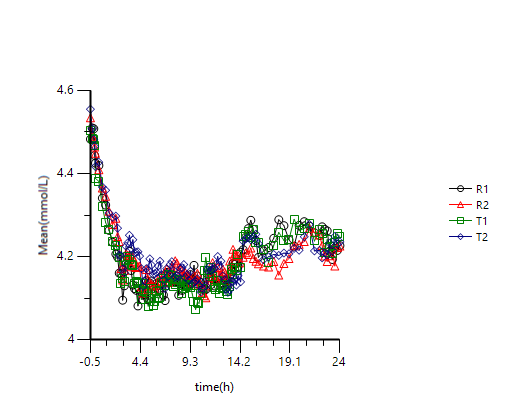


**Supplementary Figure 4.** Average blood glucose concentration-time curve of subjects before and after subcutaneous injection of test product T or reference product R.

## Supplementary Tables

Supplementary Table 1. The baseline characteristics of subjects in the TRTR group and RTRT group.

|  | TRTR | RTRT | Total |
| --- | --- | --- | --- |
| Age (years) | 28 ± 5 | 27 ± 5 | 28 ± 5 |
| Height (cm) | 170.9 ± 5.6 | 170.9 ± 4.8 | 170.9 ± 5.2 |
| Weight (kg) | 64.6 ± 5.3 | 63.6 ± 5.9 | 64.1 ± 5.6 |
| BMI (kg/m^2^) | 22.1 ± 1.0 | 21.8 ± 1.6 | 21.9 ± 1.3 |
| Blood potassium (mmol/L) | 3.88 ± 0.35 | 3.81 ± 0.31 | 3.84 ± 0.33 |

Data were presented as Mean ± SD.

Supplementary Table 2. Summary of plasma concentrations of C peptide before and after subcutaneous injection of test product T or reference product R in subjects.

|  | Basal C peptide  (ng/mL) | Average C-peptide after administration (ng/mL) | Average C-peptide after administration / Basal C peptide |
| --- | --- | --- | --- |
| Test product | 1.6 ± 0.3 (19.7) | 0.9 ± 0.3 (28.0) | 0.6 ± 0.1 (14.8) |
| Reference product | 1.6 ± 0.3 (18.9) | 0.9 ± 0.3 (32.0) | 0.6 ± 0.1 (16.1) |

Data were presented as Mean ± SD (CV%).

Supplementary Table 3. Summary of blood glucose concentrations before and after 39 subjects received test product or reference product.

|  | Basal glucose (mmol/L) | Target glucose (mmol/L) | Blood glucose after administration (mmol/L) | | Coefficient of variation of blood glucose after administration |
| --- | --- | --- | --- | --- | --- |
| Test product | 4.50 ± 0.21 (4.7) | 4.20 ± 0.21 (5.0) | | 4.18 ± 0.25 (6.0) | 6.0% |
| Reference product | 4.50 ± 0.23 (5.1) | 4.20 ± 0.23 (5.5) | | 4.18 ± 0.26 (6.3) | 6.3% |

Data were presented as Mean ± SD (CV%).

Supplementary Table 4. Summary of serious adverse events by system organ classification and preferred term.

| System organ classification (SOC) | Test product T1 (N=39) | | Test product T2 (N=36) | | Reference product R1 (N=40) | | Reference product R2 (N=38) | |
| --- | --- | --- | --- | --- | --- | --- | --- | --- |
| Preferred term (PT) | number | Subjects (%) | number | Subjects (%) | number | Subjects (%) | number | Subjects (%) |
| Serious adverse events | 1 | 1 (2.6) | 0 | 0 (0.0) | 2 | 1 (2.5) | 0 | 0 (0.0) |
| Metabolic and nutritional diseases | 0 | 0 (0.0) | 0 | 0 (0.0) | 1 | 1 (2.5) | 0 | 0 (0.0) |
| Hypokalemia | 0 | 0 (0.0) | 0 | 0 (0.0) | 1 | 1 (2.5) | 0 | 0 (0.0) |
| Infections and infectious diseases | 1 | 1 (2.6) | 0 | 0 (0.0) | 0 | 0 (0.0) | 0 | 0 (0.0) |
| Tuberculous pleurisy | 1 | 1 (2.6) | 0 | 0 (0.0) | 0 | 0 (0.0) | 0 | 0 (0.0) |
| Various neurological diseases | 0 | 0 (0.0) | 0 | 0 (0.0) | 1 | 1 (2.5) | 0 | 0 (0.0) |
| Epilepsy | 0 | 0 (0.0) | 0 | 0 (0.0) | 1 | 1 (2.5) | 0 | 0 (0.0) |

Supplementary Table 5. Summary of drug-related serious adverse events by system organ classification and preferred term.

| System organ classification (SOC) | Test product T1 (N=39) | | Test product T2 (N=36) | | Reference product R1 (N=40) | | Reference product R2 (N=38) | |
| --- | --- | --- | --- | --- | --- | --- | --- | --- |
| Preferred term (PT) | number | Subjects (%) | number | Subjects (%) | number | Subjects (%) | number | Subjects (%) |
| Drug-related Serious adverse events | 0 | 0 (0.0) | 0 | 0 (0.0) | 1 | 1 (2.5) | 0 | 0 (0.0) |
| Metabolic and nutritional diseases | 0 | 0 (0.0) | 0 | 0 (0.0) | 1 | 1 (2.5) | 0 | 0 (0.0) |
| Hypokalemia | 0 | 0 (0.0) | 0 | 0 (0.0) | 1 | 1 (2.5) | 0 | 0 (0.0) |

Supplementary Table 6. Adverse events leading to discontinuation are summarized by system organ classification and preferred term.

| System organ classification (SOC) | Test product T1 (N=39) | | Test product T2 (N=36) | | Reference product R1 (N=40) | | Reference product R2 (N=38) | |
| --- | --- | --- | --- | --- | --- | --- | --- | --- |
| Preferred term (PT) | number | Subjects (%) | number | Subjects (%) | number | Subjects (%) | number | Subjects (%) |
| Adverse events leading to discontinuation | 2 | 2 (5.1) | 0 | 0 (0.0) | 1 | 1 (2.5) | 3 | 2 (5.3) |
| Infections and infectious diseases | 2 | 2 (5.1) | 0 | 0 (0.0) | 0 | 0 (0.0) | 1 | 1 (2.6) |
| Tuberculous pleurisy | 1 | 1 (2.6) | 0 | 0 (0.0) | 0 | 0 (0.0) | 0 | 0 (0.0) |
| Device related infections | 1 | 1 (2.6) | 0 | 0 (0.0) | 0 | 0 (0.0) | 1 | 1 (2.6) |
| Various neurological diseases | 0 | 0 (0.0) | 0 | 0 (0.0) | 1 | 1 (2.5) | 0 | 0 (0.0) |
| Epilepsy | 0 | 0 (0.0) | 0 | 0 (0.0) | 1 | 1 (2.5) | 0 | 0 (0.0) |
| Vascular and Lymphatic Diseases | 0 | 0 (0.0) | 0 | 0 (0.0) | 0 | 0 (0.0) | 2 | 2 (5.3) |
| Deep vein thrombosis | 0 | 0 (0.0) | 0 | 0 (0.0) | 0 | 0 (0.0) | 1 | 1 (2.6) |
| Venous thrombosis of extremities | 0 | 0 (0.0) | 0 | 0 (0.0) | 0 | 0 (0.0) | 1 | 1 (2.6) |
